# *Metarhizium anisopliae* reshapes the citrus rhizosphere microbiome to enhance fruit quality via nutrient cycling

**DOI:** 10.3389/fpls.2026.1784405

**Published:** 2026-03-04

**Authors:** Chunxiao Han, Wen Luo, Guoxiong Peng, Demo Tan, Renlong Liu, Yueqing Cao

**Affiliations:** 1 School of Chemistry and Chemical Engineering, Chongqing University, Chongqing, China; 2 State Development & Investment Corporation (SDIC) Xinjiang Luobupo Postash Co., Ltd., Hami, China; 3 School of Life Sciences, Chongqing University, Chongqing, China; 4 Chongqing Smart Agriculture Service Group Yubei Co., Ltd., Chongqing, China

**Keywords:** *Metarhizium anisopliae*, citrus, soil microbiome, fruit quality, nutrient utilization

## Abstract

The rhizosphere microbiome is a critical regulator of nutrient acquisition and plant growth in citrus. Here, we evaluated the effects of the entomopathogenic fungus *Metarhizium anisopliae* CQMa421 on soil nutrient status, rhizosphere bacterial community structure, and fruit quality in citrus using soil physicochemical assays, plant physiological measurements, and 16S rRNA amplicon high-throughput sequencing. CQMa421 application markedly reshaped soil properties, increasing available potassium by 128.50% and organic matter by 75.05%. In addition, total nitrogen, alkali-hydrolyzable nitrogen, and available phosphorus increased by 112.68%, 155.30%, 305.74% respectively, while soil pH decreased by 0.4 units. CQMa421 treatment significantly increased leaf total nitrogen content and elevated fruit vitamin C by 12.00%. Microbial community profiling showed an enrichment of putatively beneficial taxa, including Proteobacteria and Firmicutes, in treated soils. Functional prediction suggested enhanced nutrient cycling potential, with increased representation of genes associated with carbohydrate metabolism and inorganic ion transport. Collectively, these results indicate that *M. anisopliae* CQMa421 acts as a plant growth-promoting fungus by enhancing soil nutrient availability and restructuring the rhizosphere microbiome, thereby improving the overall nutrient status of the soil and enhancing citrus fruit quality.

## 1 Introduction

Citrus (Citrus spp.) is one of the most economically important fruit crops globally, widely cultivated in tropical and subtropical regions (Zou et al., 2016). However, intensive cultivation practices in citrus production, driven by over-reliance on chemical fertilizers, have led to numerous challenges. These include nutrient imbalances, low fertilizer use efficiency, soil compaction, and alterations in microbial diversity. These problems subsequently induce nutritional disorders in trees, resulting in declines in both yield and quality (Chen et al., 2022).

Soil microbial communities play a pivotal role in regulating soil health and crop quality, with rhizosphere microbes acting as key facilitators of nutrient cycling, soil structure maintenance, and plant growth promotion (Fierer et al., 2021). In citrus cultivation, the rhizosphere microbiome significantly influences the availability of nutrients such as nitrogen and phosphorus, regulates plant growth via phytohormone production, and contributes to the suppression of plant pathogens (Elhaissoufi et al., 2021). However, intensive fertilization practices can disrupt the balance of the rhizosphere microbial community, leading to a decrease in beneficial microorganisms and, consequently, a decline in fruit quality (Ngullie et al., 2015). This underscores the need for sustainable agricultural practices that restore or enhance rhizosphere ecology, thus mitigating the negative effects of chemical fertilizers and promoting the quality of citrus fruit.

Current strategies for improving the citrus rhizosphere microecology primarily include the introduction of beneficial microbial inoculants, application of organic amendments, and implementation of ecological agricultural practices. Among these, Plant Growth-Promoting Rhizobacteria (PGPR) have demonstrated significant potential as an environmentally friendly biocontrol approach for managing citrus diseases and enhancing fruit yield. Previous studies have shown that PGPR such as *Pseudomonas* and *Bacillus* can successfully colonize citrus roots, directly promoting plant growth through phosphate solubilization, siderophore production, and phytohormone synthesis (e.g., IAA) (Bach et al., 2016), while indirectly suppressing soil-borne pathogens (e.g., *Phytophthora*) via antibiotic secretion, niche competition, and induced systemic resistance (Sivaprakasam et al., 2024). *Trichoderma*, as an important plant growth-promoting and biocontrol microorganism, particularly suppresses soil-borne pathogens like *Phytophthora*, through multiple mechanisms including mycoparasitism, antibiosis via antibiotic secretion, and competition for nutrients and space in the rhizosphere (Sivaprakasam et al., 2024).

*Metarhizium anisopliae*, a well-known entomopathogenic fungus, has gained attention for its potential to improve crop growth and control pests (Hong et al., 2017; Jiang et al., 2020; Li and Xia, 2022; Wang et al., 2026; Pria Júnior et al., 2008). While its role in general crop growth has been established, there is limited research on its specific effects in citrus cultivation. Studies have shown that *M. anisopliae* enhances soil properties, promote plant growth by secretion of bioactive compounds such as IAA and phosphate-solubilizing enzymes, and modulate the rhizosphere microbial community to favor beneficial taxa (Bamdad et al., 2022; Pan and Cai, 2023; Hu and Bidochka, 2021). Moreover, the *Metarhizium* species have been shown to promote crop growth through induced systemic resistance and modulation of the soil microbiome (Liao et al., 2017; Martins et al., 2024). Nevertheless, current understanding of the impact of *M. anisopliae* on citrus rhizosphere microecosystem and its relationship with fruit quality formation are still unclear.

In this study, we aim to investigate the effects of *M. anisopliae* CQMa421 on citrus by systematically investigating the changes of its rhizosphere soil microbial community, soil physicochemical properties, plant parameters, and fruit quality. The results can contribute valuable insights into sustainable citrus cultivation practices that reduce dependence on chemical fertilizers and promote high-quality fruit production.

## 2 Materials and methods

### 2.1 Experimental design

The experiment was conducted at Xincun Village, Yubei District, Chongqing, China (29°48′5″ N, 106°38′46″ E), a region characterized by a subtropical humid monsoon climate with distinct seasons. The highest and lowest temperatures typically occur in July and January, respectively, with an average annual precipitation of 1,156.8 mm and annual sunshine duration of 1,014.3 hours. The experimental period extended from December 2023 to December 2024. Three-year-old Satsuma mandarin (*Citrus reticulata* Blanco) was used and grown in the field. The soil in the experimental orchard was classified as Purplish soil in the Chinese Soil Taxonomy. The *M. anisopliae* strain CQMa421 used in this trial has been previously reported for insect control (Food and Agriculture Organization (FAO), 2025; Du et al., 2024).

A randomized block design was adopted in this study, comprising one experimental treatment group and one control group with three replicates each (*n* = 3). The specific treatments were as follows: Test group received compound fertilizer (12% N, 10% P, 18% K), organic fertilizer (provided by Chongqing Fengzeyuan Fertilizer Co., Ltd., containing 5% rapeseed residue and 45% fish powder), and *M. anisopliae* CQMa421 (applied at a concentration of 5 × 10^6^ conidia/mL, 5 L per plant). Control group received same fertilizer consistent with test group but without *M. anisopliae* CQMa421. Each treatment consisted of three replicate plots (50 trees per plot). For chemical and microbiological analyses, composite samples were obtained per plot and treated as independent replicates (*n* = 3). The timing and application rates for all fertilizers were as follows: basal application of organic fertilizer and compound fertilizer (12-10-18) in early January, accounting for 60% of the annual fertilizer application rate, applied through pit fertilization. *M. anisopliae* CQMa421 was applied through root irrigation. Topdressing with compound fertilizer (8-5-10) was conducted in mid-May, accounting for 40% of the annual fertilizer application rate, applied through drip irrigation with *M. anisopliae* CQMa421 application for test group as in January.

### 2.2 Sample collection

Soil samples were collected during the fruit maturation period from six trees per plot, selected from both central and peripheral areas. At each tree, two sampling points were positioned near the canopy drip line along a diagonal, avoiding fertilization trenches. After removing surface litter, a stainless-steel auger was used to collect 0–20 cm depth soil cores, with consistent sampling volume across all points. Soil from the 12 points per plot was thoroughly mixed into a composite sample, from which approximately 1 kg was taken for both treatment and control groups and transported under cool conditions to the laboratory. The fresh soil was processed in three ways: one portion stored at 4 °C for less than one week for measuring extracellular enzyme activities (sucrase, urease, phosphatase) with three independent replicates per enzyme; another portion stored at –80 °C for subsequent 16S rRNA sequencing with three independent replicates; and a third portion (500 g) air-dried, sieved through a 20-mesh screen, and used for determining soil pH and available nitrogen, phosphorus, and potassium content.

Leaf and fruit samples were collected at the same time as soil sampling. From the same citrus trees corresponding to the soil sampling points, fruits of relatively uniform size and maturity were harvested from the east, south, west, and north orientations of each tree. Every six trees constituted one biological replicate, with 24 fruits collected per replicate, resulting in a total of 72 fruits per treatment for fruit quality analysis. For leaf nutrient analysis, four healthy, mature leaves were collected from each of the upper, middle, and lower canopy layers of the same trees. Similarly, every six trees formed one replicate, with 24 leaves collected per replicate, totaling 72 leaves per treatment for leaf nutrient determination.

### 2.3 Soil physicochemical properties analysis

Soil physicochemical parameters were measured according to the methods described previously (Bao, 2000). Soil pH was measured potentiometrically in a soil-water suspension (1:2.5 w/v ratio). Soil organic matter (SOM) content was determined by the potassium dichromate oxidation method with external heating. Alkaline hydrolysis nitrogen was quantified using the alkali-diffusion method. Available phosphorus was extracted with 0.5 M NaHCO_3_ (pH 8.5) and measured by molybdenum-antimony anti-spectrophotometry. Available potassium was extracted with 1 M neutral ammonium acetate and determined by flame photometry. Sucrase activity was measured using 3,5-dinitrosalicylic acid colorimetry and expressed as mg glucose released per gram of soil after 24-hour incubation. Urease activity was determined by sodium phenolate-sodium hypochlorite colorimetry and expressed as mg NH_3_-N produced per gram of soil after 24-hour incubation. Acid phosphatase activity was assessed using disodium phenyl phosphate colorimetry and expressed as mg phenol released per gram of soil after 24-hour incubation. Additionally, total nitrogen content was determined by the semi-micro Kjeldahl method, while exchangeable calcium and magnesium were extracted with 1 M ammonium acetate and quantified by atomic absorption spectrometry. All the tests were conducted in triplicates.

### 2.4 Determination of fruit quality and leaf mineral elements

Leaf mineral elements were determined as follows after H_2_SO_4_-H_2_O_2_ digestion: nitrogen by semi-micro Kjeldahl distillation, phosphorus by molybdenum-antimony anti-spectrophotometry, potassium by flame photometry, and magnesium by atomic absorption spectrometry (Guo et al., 2023).

Fruit quality parameters were determined according to AOAC International (Horwitz and Latimer, 2010). Soluble solids content (SSC) was measured with a handheld digital refractometer (PAL-1, Atago, Japan). Vitamin C content was quantified by titration with 2,6-dichloroindophenol. Soluble sugars were determined following acid hydrolysis coupled with copper-reduction titration, whereas reducing sugars were measured using the Lane–Eynon method. Total titratable acidity was assessed by alkaline potentiometric titration. All measurements were performed in triplicate.

### 2.5 DNA extraction and sequencing

Total genomic DNA from microbial communities in soil was extracted using the YH-soil FastPure Soil DNA Isolation Kit (Magnetic bead) (MJYH, Shanghai, China) following manufacturer’s protocols. The quality of extracted genomic DNA was verified by 1% agarose gel electrophoresis, while DNA concentration and purity were determined using a NanoDrop2000 spectrophotometer (Thermo Scientific, USA).

The extracted DNA served as template for PCR amplification of the 16S rRNA gene V3-V4 hypervariable regions using barcode-indexed primers 338F (5’-ACTCCTACGGGAGGCAGCAG-3’) and 806R (5’-GGACTACHVGGGTWTCTAAT-3’). PCR products were resolved on 2% agarose gels, excised, and purified using the PCR Clean-Up Kit (Yuhua, China). Purified products were quantified with Qubit 4.0 (Thermo Fisher Scientific, USA). Sequencing libraries were prepared with the NEXTFLEX Rapid DNA-Seq Kit and sequenced on the Illumina Nextseq2000 platform (Shanghai Majorbio Bio-pharm Technology Co., Ltd.). Raw sequencing data were deposited in the NCBI SRA database (Accession: SUB15241009).

### 2.6 Bioinformatic analysis of soil bacterial communities

Raw paired-end reads were quality-filtered using fastp (v0.19.6), including trimming low-quality bases (*Q* < 20), removing reads shorter than 50 bp or containing ambiguous nucleotides. High-quality reads were merged using FLASH (v1.2.11) with a minimum overlap of 10 bp.

Denoising and amplicon sequence variant (ASV) inference were performed in QIIME2 using DADA2 with default parameters. Chloroplast and mitochondrial sequences were removed. To account for differences in sequencing depth, all samples were rarefied to 40,865 reads per sample, achieving an average Good’s coverage of 99.84%. Taxonomic classification was conducted using the QIIME2 classify-sklearn classifier against the SILVA 16S rRNA database (v138). Functional profiles were predicted using PICRUSt2 (v2.2.0).

### 2.7 Statistical analysis

All sequence data processing and microbial community analyses were performed on the Majorbio Cloud Platform (https://cloud.majorbio.com). Alpha diversity indices (Chao1 and Shannon) were calculated using mothur, and differences in alpha diversity between treatments were assessed with the Wilcoxon rank-sum test. Beta diversity was evaluated using Bray–Curtis distances and visualized by principal coordinates analysis (PCoA), and group differences in community composition were tested by PERMANOVA. Differentially abundant bacterial taxa between treatments were identified by LEfSe (Linear Discriminant Analysis Effect Size; LDA score > 2, *p* < 0.05). The relationships between soil physicochemical properties and bacterial community structure were examined using distance-based redundancy analysis (db-RDA). Species included in the co-occurrence network were selected based on Spearman correlation coefficients (|*r*| > 0.6, *p* < 0.05). Soil physicochemical properties, leaf nutrient contents, and fruit quality parameters were analyzed using one-way analysis of variance (ANOVA), with treatment plots as independent replicates (*n* = 3). When significant effects were detected, means were separated using Tukey’s HSD test. Unless otherwise stated, statistical significance was set at *p* < 0.05.

## 3 Results

### 3.1 Analysis of significant differences in soil physicochemical properties

Comparative analysis revealed significant alterations in key soil physicochemical parameters following the application of CQMa421 (**Table 1**). The CQMa421 treatment group exhibited substantial increase in soil organic matter (75.05%), total nitrogen (112.68%), alkaline hydrolyzable nitrogen (155.30%), available phosphorus (305.74%), available potassium (128.50%), compared to the control. No significant variations were observed in exchangeable magnesium (EMg) concentrations. Notably, the soil pH in the test group decreased by 0.4 units. Analysis of soil enzymatic activities revealed that the application of CQMa421 had no significant effect. These results suggest that the amendment with CQMa421 facilitated the accumulation of soil organic matter, nitrogen, and potassium, mitigated soil salinity and alkalinity, and consequently enhanced the overall content of nutrient elements in the soil.

**Table 1:** Effect of CQMa421 application on soil physicochemical properties.

Soil properties	Control group fertilizer	Test group Fertilizer + CQMa421	Change percent
pH	7.15 ± 0.00	6.75 ± 0.00	- 5.59%
Organic matter (OM, g/kg)	11.06 ± 0.22	19.36 ± 0.12	+ 75.05% ****
Total nitrogen (TN, g/kg)	0.71 ± 0.01	1.51 ± 0.01	+ 112.68% ****
Alkaline hydrolyzable nitrogen (AHN, mg/kg)	35.59 ± 0.39	90.86 ± 0.20	+ 155.30% ****
Available phosphorus (AP, mg/kg)	22.14 ± 0.60	89.83 ± 0.48	+ 305.74% ****
Available potassium (AK, mg/kg)	327.90 ± 2.23	749.24 ± 2.58	+ 128.50% ****
Exchangeable calcium (EC, cmol/kg)	17.97 ± 0.67	16.58 ± 0.69	- 7.74% ***
Exchangeable magnesium (EM, cmol/kg)	1.51 ± 0.08	1.39 ± 0.01	- 7.95%
Sucrase (U/kg)	7.12 ± 0.05	7.45 ± 0.06	+ 4.63%
Urease (U/kg)	0.07 ± 0.00	0.07 ± 0.00	0%
Acid phosphatase (U/kg)	0.20 ± 0.02	0.20 ± 0.01	0%

*p* < 0.001 marked as ***, *p* < 0.0001 marked as ****.

### 3.2 M. anisopliae promoted nutrient uptaking in citrus

**Figure 1:**
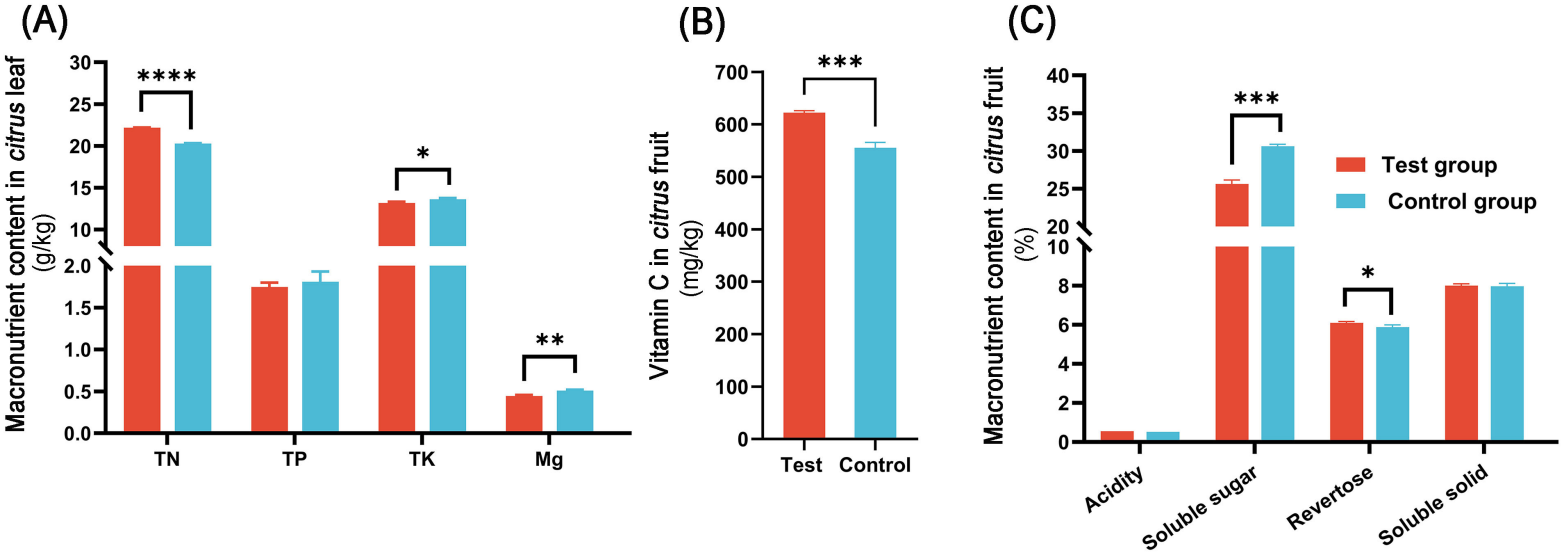
Changes in citrus plant growth and nutrient uptake under different treatments. **(A)** Nutrient status in citrus leaves; **(B)** Fruit vitamin C content; **(C)** Fruit quality parameters (TN: total nitrogen; TP: total phosphorus; TK: total potassium). *p* < 0.05 marked as *p < 0.01 marked as **p < 0.001 marked as ***p < 0.0001 marked as ****.

As shown in **Figure 1**, the application of *M. anisopliae* CQMa421 resulted in distinct alterations in leaf nutrient composition and fruit quality compared with control. In leaf tissues (**Figure 1A**), total nitrogen (TN) content in the treatment group was significantly higher than that of the control (*p* < 0.0001), increasing from 20.27 g/kg to 22.17 g/kg (a 9.37% increase). In contrast, total potassium (TK) content (*p* < 0.05) and magnesium (Mg) (*p* < 0.01) exhibited a significant reduction. No significant differences were observed in total phosphorus (TP), indicating that the CQMa421 treatment primarily influenced nitrogen assimilation.

Regarding fruit quality, fruit vitamin C content (**Figure 1B**) was markedly enhanced by CQMa421 application, increasing from 555.55 mg/kg to 622.22 mg/kg, corresponding to a 12.00% improvement (*p* < 0.001). This represents the most prominent fruit-quality enhancement among the measured traits. With respect to fruit nutritional traits (**Figure 1C**), soluble sugar content was significantly lower in the treatment group compared with the control (*p* < 0.0001). Although total acidity, revertose, and soluble solids showed slight upward trends under CQMa421 treatment, these changes were not statistically significant.

### 3.3 Soil bacterial diversity analysis

Soil samples were subjected to 16S rRNA sequencing, followed by quality control and denoising through the DADA2 plugin in the QIIME2 pipeline, which included filtering, chimera removal, and deduplication. This process yielded 10,915 amplicon sequence variants (ASVs) and 245,190 high-quality sequences. All samples were rarefied to 40,865 sequences, with a total of six samples included for subsequent analysis. The ASV table was rarefied to the minimum sequencing depth. Rarefaction curves and rank-abundance curves plateaued with increasing sequencing depth (**Supplementary Figure 1**), indicating that the sequencing effort was sufficient to capture the microbial diversity in all samples.

Bacterial alpha diversity indices are summarized in **Supplementary Table 1**. The Coverage index reached 100% for all samples, confirming adequate sampling depth. Compared to the Control group, the addition of CQMa421 (Test group) significantly increased community richness, as evidenced by higher values for the Sobs, ACE, and Chao1 indices (increases ranging between 10 and 13.28). Community diversity was also enhanced in the Test group, reflected by an increase in the Shannon index (by 0.08) and a decrease in the Simpson index. Furthermore, community evenness improved, with a higher Pielou_e value (increase of 0.01) in the Test group compared to the Control. In summary, the application of *M. anisopliae* significantly enhanced the richness, diversity, and evenness of the soil bacterial community.

### 3.4 Analysis of soil bacterial community structure

To obtain taxonomic information for each ASV, representative sequences were classified using classify-sklearn (Naïve Bayes). The results (**Supplementary Table 2**) revealed that at the phylum and class levels, the test group showed reductions of 1 phylum and 1 class compared to the control group, along with a decrease of 417 ASVs in total. In contrast, the numbers of taxa at the order, family, genus, and species levels increased by 3, 7, 30, and 35, respectively. These findings indicate that while CQMa421 suppressed certain bacterial groups at higher taxonomic levels, it significantly enhanced microbial richness at lower taxonomic ranks.

**Figure 2:**
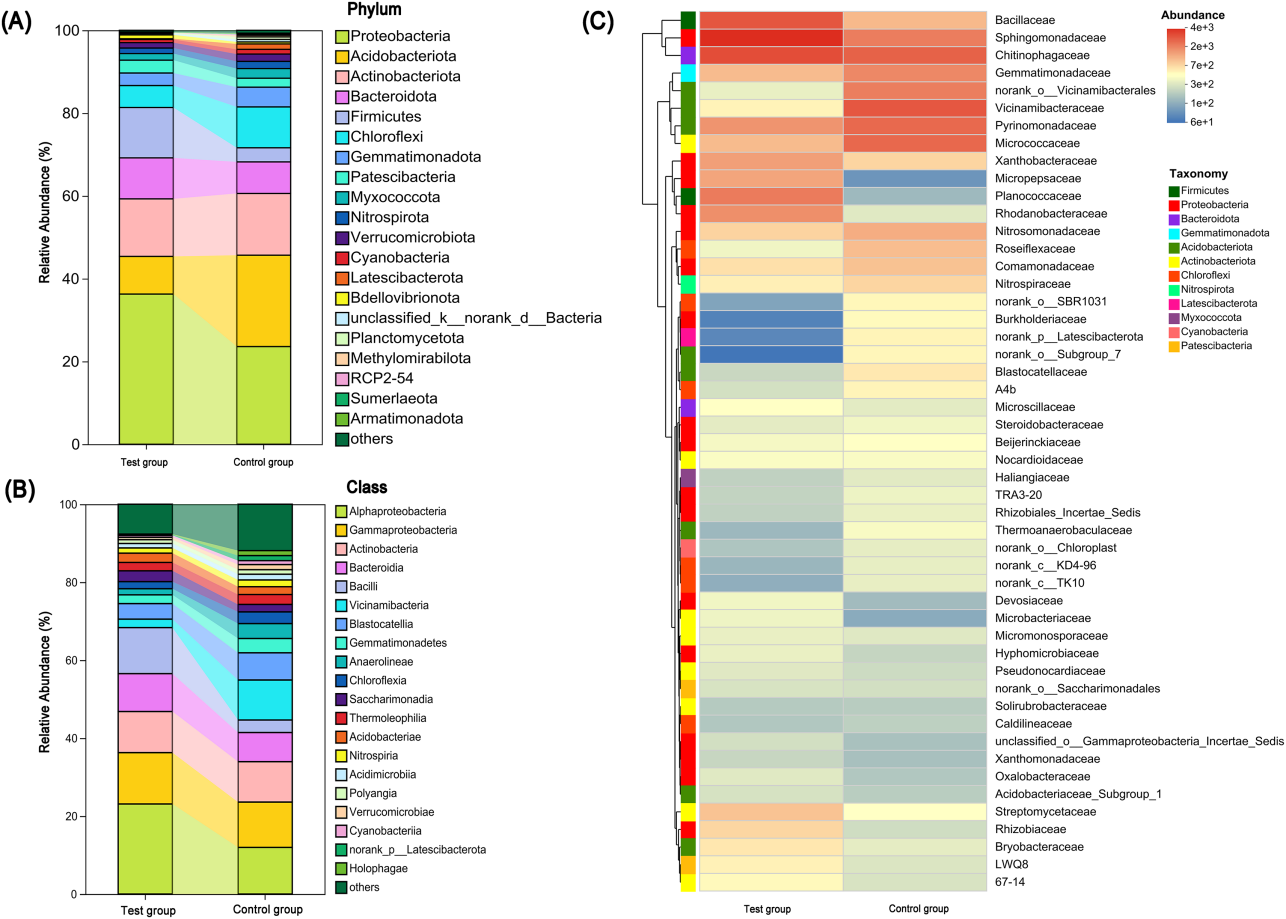
Changes in soil community structure composition. **(A)** The soil bacterial community structure at the phylum level under different fertilization treatments; **(B)** The soil bacterial community structure at the class level under different fertilization treatments; **(C)** Shared and unique species at the genus level between test group and control group.

As shown in **Figures 2A**, the citrus soil bacterial community at the phylum level was dominated by Proteobacteria, Acidobacteriota, Actinobacteriota, Bacteroidota, and Firmicutes, together accounting for more than 90% of the total relative abundance in both groups. The cumulative relative abundance of the top 10 phyla reached 95.72% in the CQMa421 treatment group and 92.52% in the control group. Compared with the control, CQMa421 supplementation significantly increased the relative abundances of Proteobacteria, Bacteroidota, and Firmicutes, while reducing those of Acidobacteriota, Actinobacteriota, Chloroflexi, and Gemmatimonadota.

At the class level (**Figure 2B**), Alphaproteobacteria, Gammaproteobacteria, Actinobacteria, Bacteroidia, and Bacilli were the predominant taxa across both treatments. The CQMa421 treatment significantly enriched copiotrophic classes such as Alphaproteobacteria, Gammaproteobacteria, and Bacilli, while reducing the relative abundance of oligotrophic or slow-growing groups, including Vicinamibacteria, Blastocatellia, Anaerolineae, and Gemmatimonadetes. In addition, shifts in community composition involved the appearance and disappearance of several low-abundance classes, mainly affiliated with the Gemmatimonadota, Chloroflexi, Acidobacteriota, and Planctomycetota phyla.

Consistent with these taxonomic shifts, hierarchical clustering analysis based on the top 50 bacterial families (**Supplementary Figure 2**) revealed clear differentiation between the two groups. The control soils were primarily characterized by Sphingomonadaceae, Chitinophagaceae, and Gemmatimonadaceae, whereas the CQMa421 treatment soils were enriched in Bacillaceae, Rhodanobacteraceae, and Sphingomonadaceae. Overall, CQMa421 application resulted in a marked restructuring of the soil bacterial community, as reflected by substantial gains and losses in family-level taxa.

At the genus level (**Figure 2C**), CQMa421 treatment resulted in the addition of 155 genera and the reduction of 125 genera. Notably enriched genera included *Bacillus*, *Phreatobacter*, and *Sporosarcina*, predominantly affiliated with the phyla Firmicutes and Proteobacteria. In contrast, the decreased genera were largely associated with Acidobacteriota and Actinobacteriota. Among the shared genera across treatments, the relative abundances of *Sphingomonas*, *Bacillus*, and *Sporosarcina* were significantly elevated, while those of *RB41* and several unclassified taxa demonstrated notable declines.

### 3.5 Analysis of differential soil bacterial taxa under different treatments

Principal coordinates analysis (PCoA) based on Bray-Curtis distances revealed that the soil bacterial community structures of the two treatment groups exhibited both similarities and distinct separations (**Supplementary Figure 3**). The first principal coordinate (PCoA1) and the second principal coordinate (PCoA2) explained 76.88% and 10.09% of the community variation, respectively. Collectively, these two components accounted for 59.01% of the observed differences in bacterial community structure.

**Figure 3:**
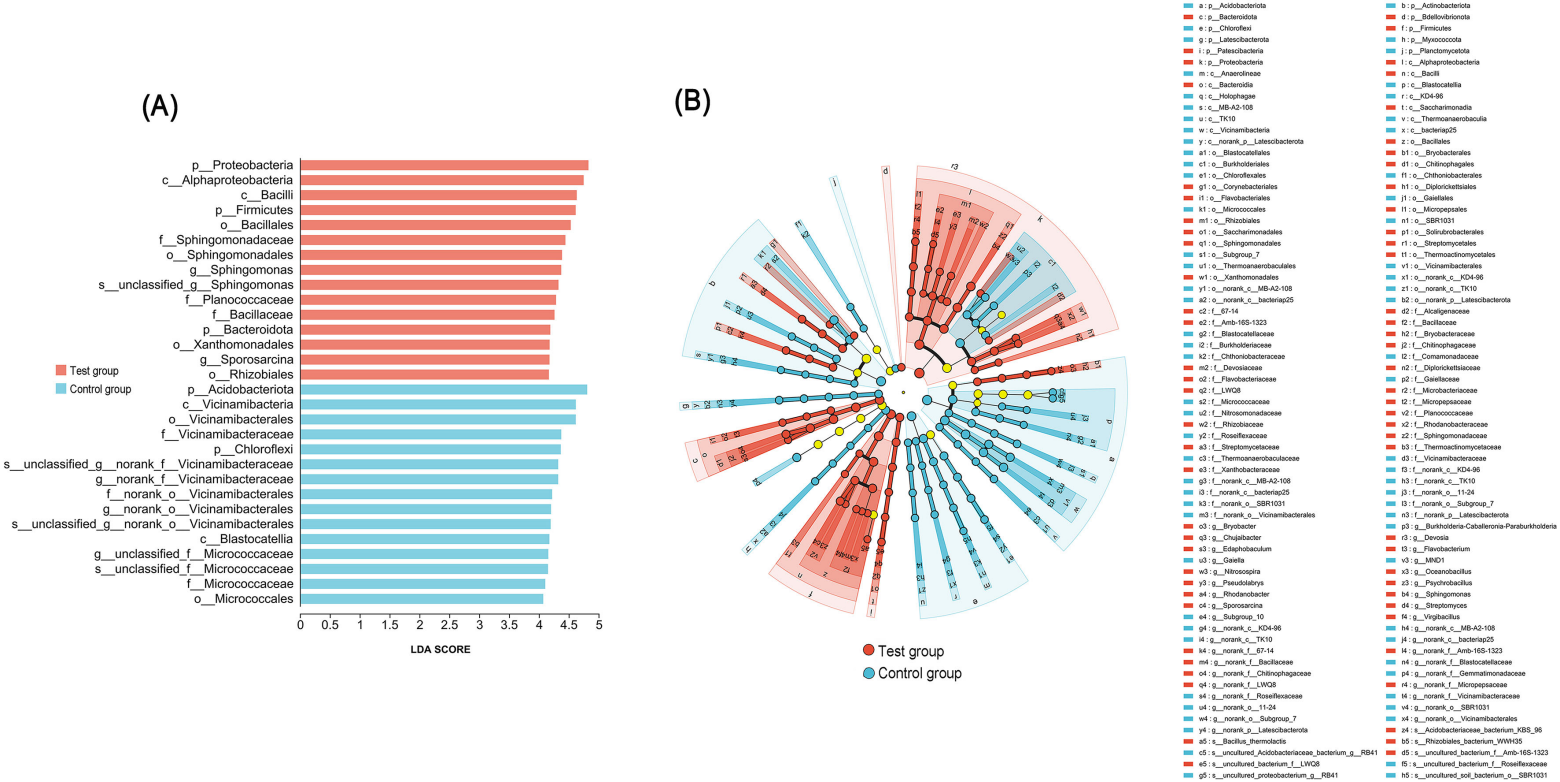
Analysis of differential soil bacterial taxa under different treatments. **(A)** shows the bar plot of bacterial biomarkers; **(B)** The phylogenetic distribution of bacterial taxa.

LEfSe analysis (LDA > 3.3, *p* < 0.05) identified a total of 138 bacterial taxa exhibiting significant differences between the two treatments (**Figures 3A, B**). In the test group amended with CQMa421, the significantly enriched taxa included the phylum Proteobacteria, class Alphaproteobacteria, class Bacilli, phylum Firmicutes, order Bacillales, family Sphingomonadaceae, genus *Sphingomonas*, family Planococcaceae, family Bacillaceae, phylum Bacteroidota, order Xanthomonadales, genus *Sporosarcina*, and order Rhizobiales. In contrast, the control group receiving only compound fertilizer showed significantly enriched indicator taxa primarily comprising the phylum Acidobacteriota, class Vicinamibacteria, order Vicinamibacterales, family Vicinamibacteraceae, phylum Chloroflexi, class Blastocatellia, and family Micrococcaceae.

### 3.6 Functional shifts in soil microbial communities

**Figure 4:**
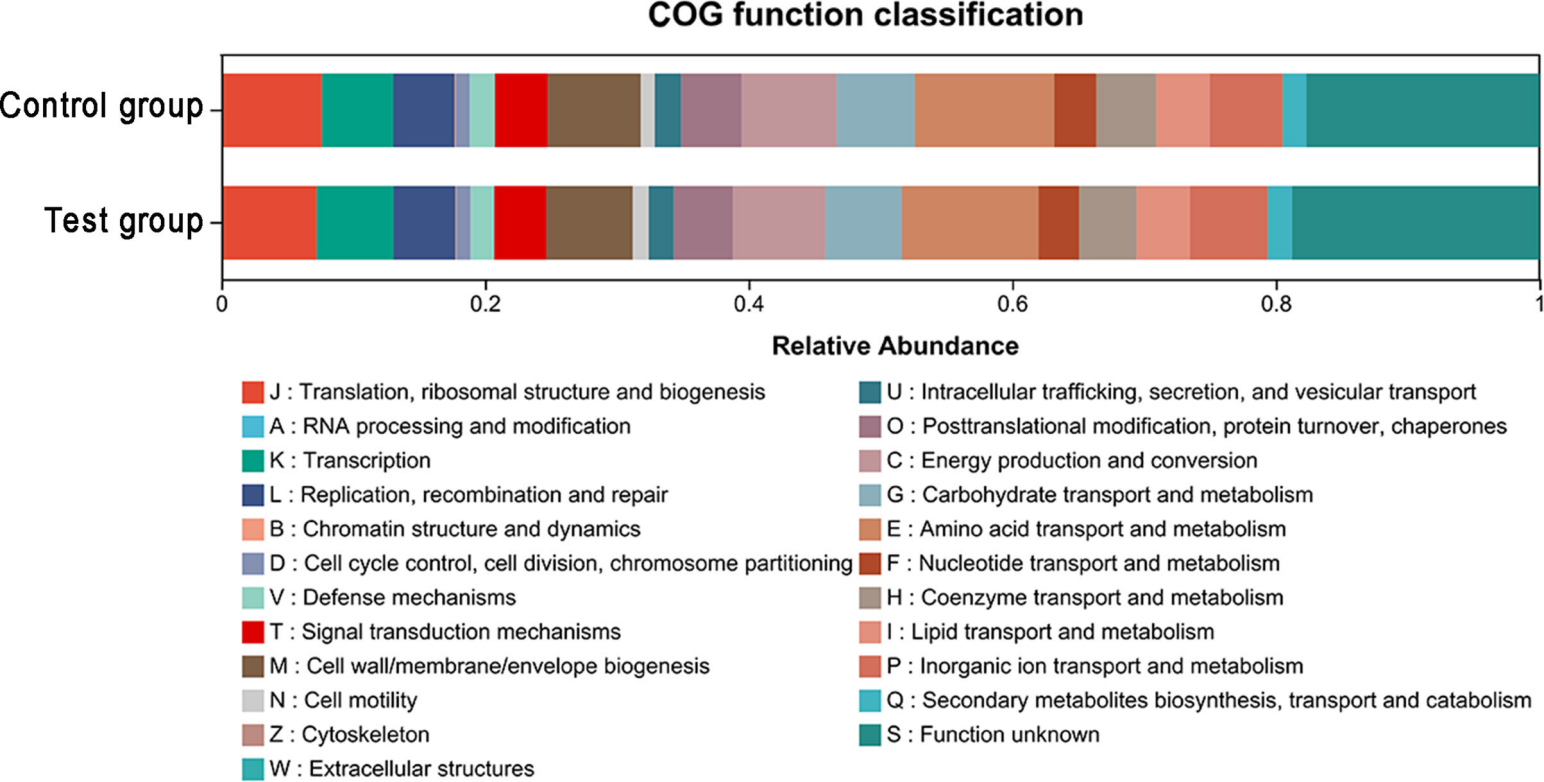
Functional distribution of rhizosphere bacteria in citrus soil.

As shown in **Figure 4**, COG functional annotation revealed clear differences in the functional profiles of citrus rhizosphere microbial communities among treatments. Compared with the control, the CQMa421 treatment exhibited significantly higher relative abundances of genes associated with transcription, DNA replication, recombination and repair, cell motility, lipid transport and metabolism, inorganic ion transport and metabolism, and the biosynthesis, transport and catabolism of secondary metabolites. The enrichment of these functional categories suggests an enhanced potential for microbial metabolic activity, cellular maintenance, and resource acquisition, which may contribute to improved adaptability of rhizosphere microorganisms to soil environmental conditions.

In addition, the relative abundance of the COG category “Function unknown” (Category S) was higher in the treatment group than in the control. This increase indicates the possible involvement of uncharacterized genes or functional pathways in microbial responses to the application of CQMa421. Such changes highlight the functional flexibility of the rhizosphere microbial community and its capacity to adjust to fertilization practices. These findings also point to the presence of unexplored microbial functional potential in the citrus rhizosphere, providing a basis for future studies aimed at elucidating microbial contributions to nutrient utilization and stress tolerance in citrus production systems.

### 3.7 Soil bacterial co-occurrence network analysis under fungal treatments

**Figure 5:**
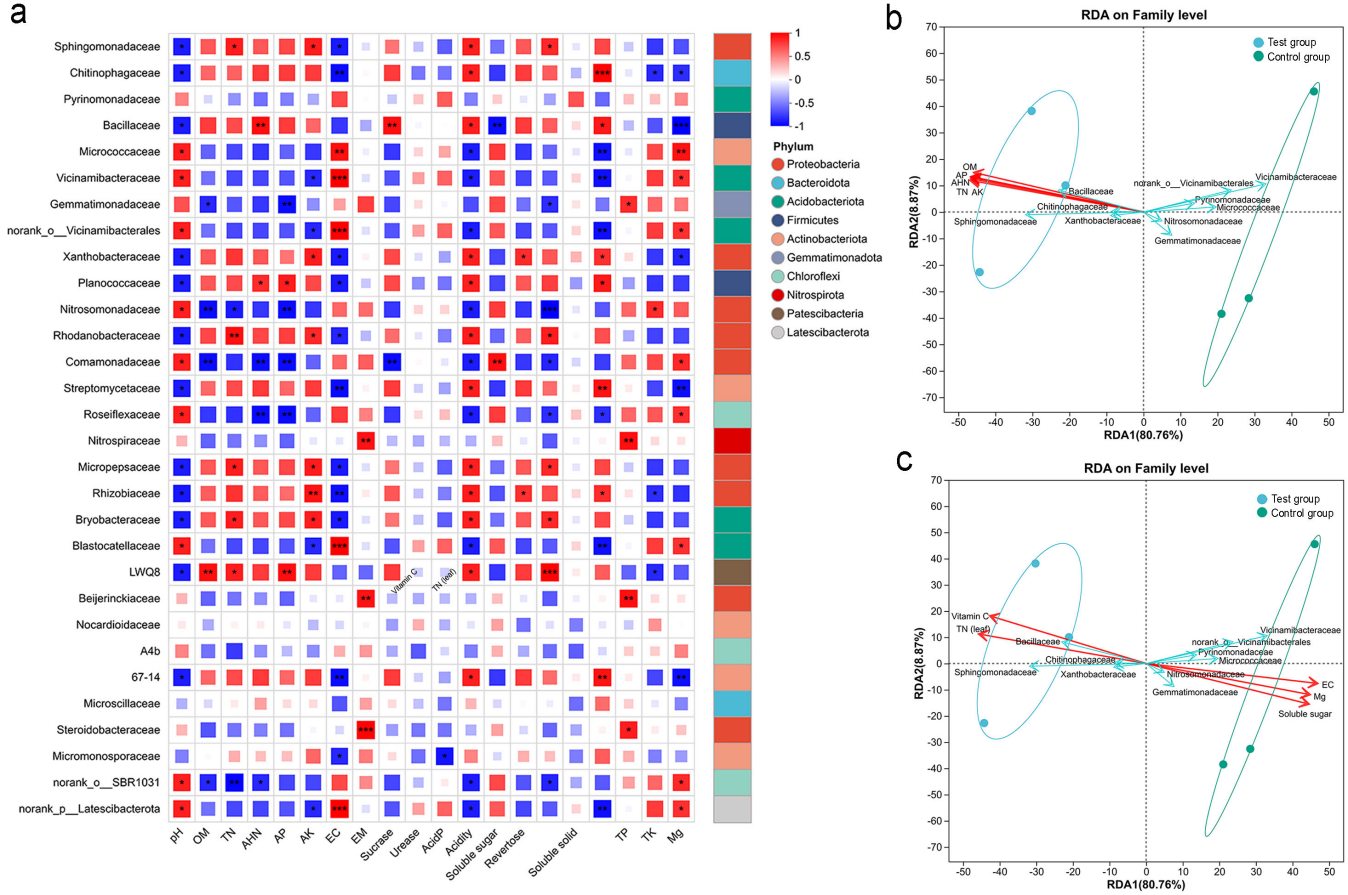
Associations among rhizosphere bacterial communities, soil properties, and citrus plant performance. **(A)** Association heatmap network shows significant relationships between dominant bacterial taxa and soil physicochemical properties, leaf nutrient contents, and fruit quality parameters. *R* values are represented by gradients of red and blue indicating positive and negative correlations, respectively. Asterisks denote significant differences (*p < 0.05, **p < 0.01, ***p < 0.001); **(B, C)** redundancy analysis (RDA) of soil physicochemical properties, citrus fruit quality parameters, leaf nutrient contents, and soil microbial communities at *p* ≤ 0.001.

As shown in **Figures 5A**, microbial–environmental correlation network analysis indicated that CQMa421 application markedly altered the associations between dominant bacterial taxa and soil and plant variables. Several bacterial families, including Planococcaceae, Sphingomonadaceae, Bacillaceae, Micropepsaceae, *LWQ8*, Rhodanobacteraceae, and Bryobacteraceae, exhibited strong positive correlations (*R* > 0.7) with soil total nitrogen (TN) and available phosphorus (AP). In addition, Bacillaceae and Nocardioidaceae were highly correlated with alkaline hydrolyzable nitrogen (AHN) (*R* > 0.85), suggesting their close association with nitrogen transformation processes under CQMa421 application.

Soil physicochemical properties were also closely linked to shifts in microbial composition. Soil pH decreased by approximately 0.4 units following CQMa421 treatment and was strongly correlated with several bacterial families, including Vicinamibacteraceae, Nitrosomonadaceae, Micrococcaceae, Comamonadaceae, Blastocatellaceae, and Roseiflexaceae (*R* > 0.87). Available potassium (AK) increased markedly compared with the control and showed significant positive correlations with Sphingomonadaceae, Micropepsaceae, Rhizobiaceae, Xanthobacteraceae, and Bryobacteraceae (*R* > 0.88). Soil organic matter (OM) content was positively correlated with *LWQ8* (*R* = 0.94) (**Figure 5A**).

Microbial taxa were further associated with plant nutrient status and fruit quality parameters. Several families, including Planococcaceae, Bacillaceae, Streptomycetaceae, and 67-14, were positively correlated with leaf total nitrogen, while Beijerinckiaceae and Nitrospiraceae were associated with leaf phosphorus content. Nitrosomonadaceae and Micrococcaceae were correlated with leaf potassium and magnesium contents, respectively. In terms of fruit quality, Sphingomonadaceae, Micropepsaceae, LWQ8, Rhodanobacteraceae, and Bryobacteraceae showed positive correlations with vitamin C and total acid contents, whereas Rhizobiaceae, Xanthobacteraceae, and Comamonadaceae were associated with sugar-related traits. Notably, these microbial–environmental associations were absent in the control group.

The microbial–enzyme activity network was also modified by CQMa421 treatment. Sucrase activity was negatively correlated with Comamonadaceae (*R* = −0.94), while showed a positive correlation with Bacillaceae (*R* = 0.94), indicating potential shifts in microbial contributions to soil nutrient transformation (**Figure 5A**).

Based on the RDA results (**Figures 5B, C**), treatment with CQMa421 was the primary driver of variation in the citrus soil microbial community. The first constrained axis (RDA1) explained 80.76% of the total variance and clearly separated the treatment group (left side) from the control group (right side). Soil nutrient indicators (OM, AP, AHN, TN, AK) were all positively correlated with the treatment group (**Figure 5B**). In contrast, plant-fruit traits showed a divergent pattern: fruit vitamin C and leaf total nitrogen were positively associated with the treatment group, whereas soil exchangeable calcium, leaf magnesium and fruit soluble sugar were positively associated with the control group (**Figure 5C**). At the family level, bacterial taxa also separated into two distinct clusters. Taxa positively correlated with the CQMa421 group, in descending order of association strength, were Sphingomonadaceae, Bacillaceae, Xanthobacteraceae and Chitinophagaceae. Those positively correlated with the control group, were Vicinamibacteraceae, norank_o_Vicinamibacterales, Micrococcaceae, Pyrinomonadaceae, Gemmatimonadaceae and Nitrosomonadaceae.

## 4 Discussion

In this study, we demonstrated that the application of Metarhizium anisopliae CQMa421 reshaped the rhizosphere microbiome, leading to improved soil nutrient status and enhanced fruit quality. This was primarily driven by changes in nutrient cycling facilitated by beneficial microbial taxa, which subsequently impacted the plant’s nutrient uptake and the quality of citrus fruits, such as the significant increase in vitamin C content. These results indicate that *M. anisopliae* CQMa421 functions as a key ecological regulator linking soil microbiome restructuring with plant nutrient uptake and quality formation, consistent with emerging microbiome–plant interaction frameworks (Trivedi et al., 2020).

### 4.1 M. anisopliae restructures the soil microbial community

Application of *M. anisopliae* CQMa421 markedly altered the structure and diversity of the citrus rhizosphere microbiome. Increased alpha diversity indices indicated enhanced microbial richness and evenness, while taxonomic shifts favored copiotrophic and metabolically active phyla (Proteobacteria and Bacteroidota) and suppressed oligotrophic or acid-sensitive groups (Acidobacteriota and Actinobacteriota), a pattern commonly associated with improved nutrient availability (Xu et al., 2018; Wu et al., 2024).

These changes are likely mediated by a combination of direct and indirect fungal effects. Bioactive metabolites secreted by *Metarhizium*, including phytohormones and nutrient-solubilizing enzymes, may selectively promote beneficial bacterial taxa such as Bacillaceae while inhibiting sensitive groups (Liao et al., 2017). In parallel, fungal colonization may modify root exudate composition and soil physicochemical properties (e.g., reduced pH and increased organic matter), thereby creating a microenvironment conducive to functional microbial succession (Xu et al., 2018; Wu et al., 2024). Consistently, enrichment of plant growth–promoting taxa (Rhizobiaceae, Sphingomonas, Pseudomonadaceae, and Burkholderiaceae) and suppression of potential pathogens suggest that *M. anisopliae* enhances both nutrient acquisition and rhizosphere health (Trivedi et al., 2010; Lombardo et al., 2024).

### 4.2 Regulation of soil nutrient cycling by M. anisopliae CQMa421

*M. anisopliae* CQMa421 exerted multi-level regulatory effects on soil nutrient cycling, with significant increases in soil nitrogen fractions and other available nutrients, which are consistent with previous findings showing that *Metarhizium* can function as a rhizosphere-competent fungus, enhancing nutrient cycling and availability through nitrogen translocation, organic acid production, and modulation of rhizosphere microbial communities (Barelli et al., 2016; González-Pérez et al., 2022). The concurrent decrease in soil pH further facilitated mineral dissolution while selectively favoring acid-tolerant functional taxa such as Proteobacteria (Xu et al., 2018). At the functional level, enrichment of inorganic ion transporter genes indicates intensified microbe-mediated mineral ion turnover. Although some mineral ions, such as potassium content, showed a declining trend in leaf despite increased soil availability. This paradox may reflect dynamic redistribution of potassium within the plant, a process influenced by potassium translocation to vacuoles or storage organs rather than being directly allocated to the leaves. Furthermore, microbial immobilization could have played a role, where potassium was temporarily sequestered by microbes in an unavailable form, reducing its immediate availability for the plant (Wu et al., 2024; Pérez-Piqueres et al., 2025).

### 4.3 Microbe–plant interactions underlying improved nutrient uptake and fruit quality

From a physiological perspective, *M. anisopliae* appears to enhance fruit quality primarily through the regulation of plant nutrient acquisition, coordinated hormonal effects, and microbe-mediated protection against oxidative stress. Increasing evidence indicates that *M. anisopliae* can function as a beneficial rhizosphere or endophytic fungus, improving plant nitrogen uptake efficiency by modifying root architecture, stimulating rhizosphere microbial activity, and enhancing nitrogen transformation processes in soil (Barelli et al., 2016; Glick, 2012). The increased leaf nitrogen content observed in this study likely reflects improved nitrogen acquisition and assimilation, which supports photosynthetic capacity and assimilate production.

In parallel, fungal-derived phytohormones such as indole-3-acetic acid and gibberellins may promote root development and facilitate nutrient transport within the plant (Barelli et al., 2016; Zhao et al., 2021). Although potassium-mediated regulation of sucrose metabolism, particularly via key enzymes such as SPS and SS, may also contribute to fruit sugar accumulation (Wu et al., 2024), nitrogen availability appears to play a central role in shaping leaf nutritional status. In addition, bioactive compounds produced by functional microbial communities may alleviate oxidative stress and maintain cellular integrity under field conditions, further supporting nutrient assimilation and fruit development (Yang et al., 2021).

## 5 Summary

Overall, this study demonstrates that *M. anisopliae* CQMa421 acts as a key ecological regulator by restructuring the rhizosphere microbiome, optimizing nutrient cycling, and enhancing fruit quality. These results offer valuable mechanistic insights into how microbial community shifts directly influence nutrient acquisition and fruit quality formation, providing a novel approach to improving citrus production in a sustainable manner.

## Data Availability

The datasets presented in this study can be found in online repositories. The names of the repository/repositories and accession number(s) can be found below: https://www.ncbi.nlm.nih.gov/genbank/, PRJNA1248214.
