# Supplementary material for: Metarhizium anisopliae reshapes the citrus rhizosphere microbiome to enhance fruit quality via nutrient cycling
Source: Front Plant Sci. 2026 Mar 4;17:1784405. doi: 10.3389/fpls.2026.1784405 (PMC12996125; doi:10.3389/fpls.2026.1784405)
Supplement: Supplementary file 1 [file DataSheet1.docx]

**Article title:** *Metarhizium anisopliae* improves citrus fruit quality and nutrient uptake via remodeling of the rhizosphere microbiome

**Table S1. Bacterial alpha diversity indices**

| Samples  Estimators | Sobs | ACE | Chao | Shannon | Simpson | Coverage | Pielou_e |
| --- | --- | --- | --- | --- | --- | --- | --- |
| Test group | 482.00 ± 7.55 | 485.66 ± 10.22 | 484.08 ± 9.47 | 4.96 ± 0.03 | 0.016 ± 0.00 | 1.00 ± 0.00 | 0.80 ± 0.01 |
| Control group | 472.00 ±11.15 | 472.38 ± 11.25 | 472.05 ± 11.18 | 4.88 ± 0.07 | 0.018 ± 0.00 | 1.00 ± 0.00 | 0.79 ± 0.01 |

**Table S2.** **Taxonomic classification statistics of bacterial species**

| Samples  Kind | Kingdom | Phylum | Class | Order | Family | Genus | Species | ASV |
| --- | --- | --- | --- | --- | --- | --- | --- | --- |
| Test group | 1 | 33 | 101 | 232 | 359 | 654 | 1181 | 3935 |
| Control group | 1 | 34 | 102 | 229 | 352 | 624 | 1146 | 4352 |

****
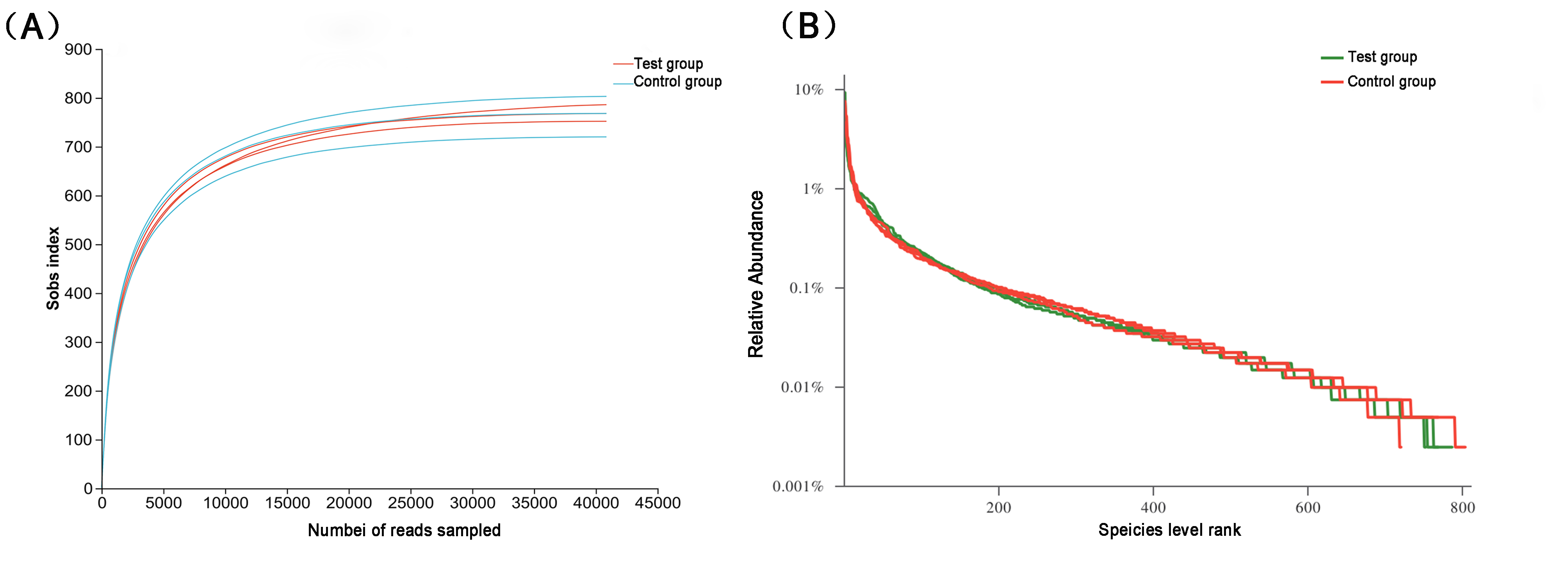
****

****Fig. S1**** (A). Rarefaction curve based on observed species (Sobs index); (B). Rank-abundance curve.

**
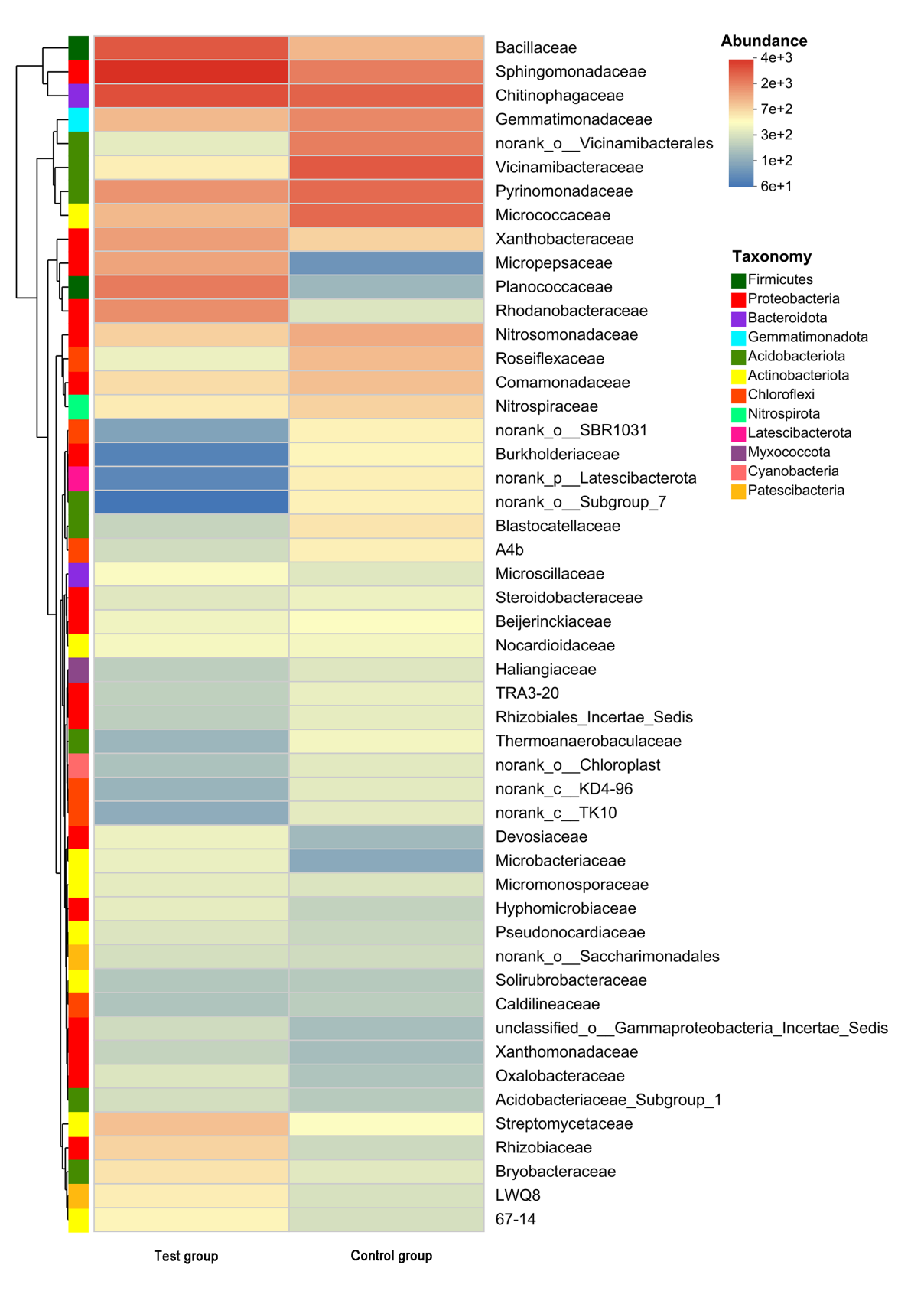
Fig. S2** Changes in soil community structure composition at the family level


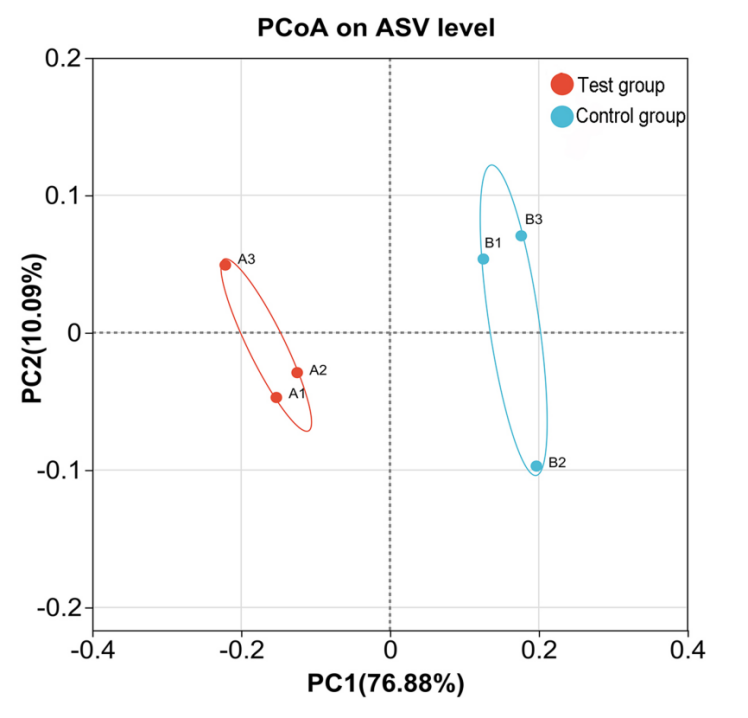


**Fig. S3** The PCoA diversity analysis of bacterial communities
